# Understanding Individual Differences in Domain-General Prosociality: A Resting EEG Study

**DOI:** 10.1007/s10548-018-0679-y

**Published:** 2018-09-28

**Authors:** Lorena R. R. Gianotti, Franziska M. Dahinden, Thomas Baumgartner, Daria Knoch

**Affiliations:** Department of Social Psychology and Social Neuroscience, Institute of Psychology, University of Bern, Fabrikstrasse 8, 3012 Bern, Switzerland

**Keywords:** Domain-general prosociality, Neural trait, Resting electroencephalography, Temporo-parietal junction

## Abstract

Prosocial behavior is of vital importance for the smooth functioning of society. However, the propensity to behave in a prosocial manner is characterized by vast individual differences. In order to reveal the sources of these differences, some studies have used objective, task-independent neural traits, for instance resting electroencephalography (EEG). Despite providing valuable insights into the neural signatures of several domains of prosociality, each of these studies has only focused on one single domain. Here, we exposed 137 participants to different social dilemma situations in order to obtain a measure of the individuals’ *domain-general* prosociality and recorded multi-channel task-independent, resting EEG. Using a source-localization technique, we found that resting current density within the temporo-parietal junction in two beta bands (beta2 and beta3) was positively associated with domain-general prosociality. This is the first demonstration of neural signatures underlying individual differences in the propensity to behave in a prosocial manner across different social situations.

## Introduction

The ability to behave in a prosocial manner is a prerequisite for large-scale social living. However, prosocial behavior is characterized by vast inter-individual heterogeneity (Kurzban and Houser 2001; Andreoni and Miller 2002; Fischbacher and Gächter 2010). Attempts to investigate the origins of this heterogeneity via personality questionnaires have been only moderately successful (e.g., Becker et al. 2012; Epstein et al. 2016). One reason for this might lie in the fact that self-reporting is susceptible to various sources of bias, including socially desirable responding, random responding, and demand characteristics (Edwards 1957; Nichols and Maner 2008). As an alternative, some studies in the fields of social neuroscience and neuroeconomics applied a so-called neural trait approach, which allows for objective and stable measurement of dispositional differences (for a review, see Nash et al. 2015). Neural traits are much like neural ‘fingerprints’ and have been used to reveal sources of individual differences in various aspects of prosociality, such as altruism (Morishima et al. 2012), cooperation (Fermin et al. 2016), costly punishment (Knoch et al. 2010), honesty (Baumgartner et al. 2013), or reciprocity (Watanabe et al. 2014).

Although these studies have provided valuable insights into the neural signatures of several domains of prosociality, each of them has only focused on one single domain. However, as previous behavioral studies have documented the existence of a domain-general prosocial ‘phenotype’ (e.g., Peysakhovich et al. 2014), it is important to investigate the neural signatures underlying prosocial behavior in more than one single domain of prosociality.

Here, we recorded task-independent resting electroencephalogram (EEG) of 137 participants before they were subjected to different social dilemma situations. Social dilemma situations have been extensively used to model complex social interactions and allow for rigorous empirical investigations. Each of the situations used in this study was characterized by a unique incentive structure, and participants were required to allocate money amongst themselves and other participants. In order to measure domain-general prosociality, which is the common denominator of prosocial behavior across the different social situations, we applied a factor analysis.

Task-independent EEG at rest constitutes an ideal neural trait measure due to its high specificity (i.e., the extent to which an EEG pattern is uniquely associated with a given person; Dünki et al. 2000; Näpflin et al. 2007) and high stability over time (e.g., Dünki et al. 2000; Näpflin et al. 2007; Cannon et al. 2012). In addition, it has been used to reveal sources of individual differences in time preferences (Gianotti et al. 2012), risk preferences (Gianotti et al. 2009; Studer et al. 2013), and social preferences (Knoch et al. 2010; Baumgartner et al. 2013; for a review, see; Nash et al. 2015). Hence, resting EEG is a promising tool to investigate possible sources of individual differences in domain-general prosociality.

Given that this was the first study of its kind, we conducted exploratory whole-brain-corrected analyses without any a-priori hypotheses to investigate the neural signatures of individual differences in domain-general prosociality.

## Materials and Methods

### Participants

We collected data from 137 (105 female) right-handed, German-speaking participants, recruited at the University of Bern. All participants indicated that they had no current or previous history of neurological or psychiatric disorders or alcohol or drug abuse. Four participants were excluded from the analyses due to technical problems during the EEG or behavioral recordings. The mean age of the remaining 133 participants (102 female) was 21 years (SD = 3). The study was approved by the local ethics committee. All participants provided written, informed consent and were informed of their right to discontinue participation at any time. Participants were remunerated with a flat fee of 40 Swiss francs (CHF 1 ≈ USD 1) in addition to the money earned in the social dilemma situations. We recruited participants for 1 academic year and collected as much data as possible during that time. Data was collected in a single wave and then analyzed after the testing was complete. Data are available upon request.

### Procedure

The electroencephalographic and behavioral data collections were separated by several weeks. First, participants completed the EEG recording in our EEG laboratory. After providing written informed consent, participants completed the Positive and Negative Affect Schedule (PANAS, Watson et al. 1988) and a handedness inventory (Chapman and Chapman 1987). Participants were seated in a sound-attenuating, electrically shielded chamber with dim illumination and an intercom connection to the experimenters. Participants were instructed that the EEG recording was to be conducted while they rested with their eyes alternately open or closed. The resting EEG protocol consisted of the participants resting for 20 s with their eyes open, followed by 40 s with their eyes closed; this was repeated five times. The instructions regarding eye opening/closing were provided via the intercom. Data analysis was based on the 200 s eyes-closed condition.

Participants were then subjected to four social dilemma situations in our behavioral laboratory with 24 interconnected computer terminals. Moreover, participants filled out the Honesty-Humility scale of the HEXACO questionnaire (Ashton and Lee 2009), which has been associated with individual differences in prosociality (Hilbig et al. 2012; Aghababaei et al. 2014; Thielmann and Hilbig 2014).

### EEG Recording and Pre-processing

Resting EEG was continuously recorded using 60 Ag–AgCl electrodes mounted in an elastic cap and placed according to the international 10–10 system (Nuwer et al. 1998). The electrode at position FCz was the recording reference, while the electrode at position CPz served as the ground electrode. Data were recorded at a sampling rate of 500 Hz (bandwidth: 0.1–250 Hz). Horizontal electrooculographic (EOG) signals were recorded at the left and right outer canthi, and vertical EOGs were recorded below the right eye. Impedances were maintained at < 10 kΩ. Eye-movement artifacts were removed using independent component analysis. After an automatic artifact rejection (maximal allowed voltage step: 15 µV; maximal allowed amplitude: ± 100 µV; minimal allowed activity in intervals of 100 ms: 0.5 µV), data were visually inspected to eliminate residual artifacts. The data were then recomputed against the average reference. All artifact-free 2 s epochs were extracted. On average, 87.1 epochs (SD = 16.7) per participant were eventually available. A Fast Fourier Transformation (using a square window) was applied to each epoch and channel to compute the power spectra with 0.5 Hz resolution. The spectra for each channel were averaged over all epochs for each participant. Absolute power values were integrated for the following seven independent frequency bands (Kubicki et al. 1979): delta (1.5–6 Hz), theta (6.5–8 Hz), alpha1 (8.5–10 Hz), alpha2 (10.5–12 Hz), beta1 (12.5–18 Hz), beta2 (18.5–21 Hz), and beta3 (21.5–30 Hz).

Standardized low-resolution electromagnetic tomography (sLORETA; Pascual-Marqui 2002) was used to estimate the intracerebral electrical sources that generated the scalp-recorded activity at each of the EEG frequency bands. The sLORETA method is a properly standardized, discrete, 3D distributed, linear, minimum norm inverse solution that allows for localization of the intracerebral sources of scalp-recorded electromagnetic signals. The particular form of standardization used in sLORETA endows the tomography with the property of exact localization to test point sources, and thereby yields images of standardized current density with exact localization, albeit with low spatial resolution (i.e., neighboring neural sources will be highly correlated). sLORETA has been validated in several simultaneous EEG/fMRI studies (Mobascher et al. 2009a, b) and in an EEG localization study for epilepsy (Rullmann et al. 2009). In the current implementation of sLORETA, computations are conducted in a realistic head model using the MNI152 template (Mazziotta et al. 2001), with the 3D solution space restricted to cortical gray matter, as determined by the probabilistic Talairach atlas (Lancaster et al. 2000). The intracerebral volume is partitioned in 6239 voxels at 5 mm spatial resolution. Thus, sLORETA images represent the standardized electric activity at each voxel in neuroanatomic Montreal Neurological Institute (MNI) space as the exact magnitude of the estimated current density. Using the automatic regularization method in the sLORETA software, we selected the transformation matrix with the signal-to-noise ratio set to ten. To reduce confounds that have no regional specificity, for each participant, sLORETA images were normalized to a total power of one and then log-transformed before statistical analyses. Due to the higher number of female participants, we first regressed the putative sex-influence out of the sLORETA images. The standardized sLORETA residuals were then used for all further analyses.

### Measurements of Domain-General Prosociality

A total of nine behavioral sessions were conducted with an average number of 16 participants per session. At the beginning of each session, participants were randomly assigned to cubicles where they made their decisions with complete anonymity from the other participants. They were not allowed to talk to each other. Participants were confronted with four social dilemma situations (see below) that measured two domains of prosociality: cooperation (public goods game; PGG) and generosity (dictator game; DG). Participants played one trial in each of the four social dilemma situations. Control questions ensured participants’ understanding of the social dilemma situations. There was no time constraint when participants made their decisions. Participants received feedback on the other participants’ choices at the end of the experiment.

### Situation 1 (PGG-2)

Participants were assigned to a group of four. Each participant was then endowed with 20 monetary units (1 MU = 0.5 CHF, 20 MUs = 10 CHF) and asked to indicate how many MUs they were willing to contribute to a public good (one-shot). Each contributed MU was multiplied by 2 and split equally among the four group members. A participant’s earning consisted of all the MUs earned from the public good as well as the MUs not contributed in the first place. After their contribution decision, we asked participants how many MUs they believed others had contributed on average.

### Situation 2 (PGG-1.2)

The group size and initial endowment were the same as in the first social dilemma situation. However, here each MU contributed to the public good (one-shot) was multiplied by 1.2. Thus, if a participant contributed 20 MUs to the public good and the other three players did not contribute at all, the monetary consequences for this participant were worse in this situation (a reduction of 14 MUs) than in Situation 1 (namely a reduction of 10 MUs). Hence, a prosocial contribution decision in Situation 2 indicated a higher level of prosociality than the same contribution decision in Situation 1. As in Situation 1, after their contribution decisions, we asked participants how many MUs they believed others had contributed on average.

### Situation 3 (PGG-3.2)

As in Situation 2, group size and initial endowment remained the same. However, here each MU contributed to the public good (one-shot) was multiplied by 3.2. In the example of a participant who contributed 20 MUs to the public good while the other three players did not contribute at all, the monetary consequences for this participant were less severe (namely a reduction of the initial endowment by 4 MUs) than those in both previous situations (Situation 1: reduction of 10 MUs; Situation 2: reduction of 14 MUs). Thus, in this situation, contributing was minimally costly and maximally beneficial to others, while in Situation 2, contributing was maximally costly and minimally beneficial to others. In Situation 1, the costs and benefits were in between those of the latter situations. That is, the same contribution was—by definition—more ‘expensive’ when the multiplier (i.e., the number by which each point contributed to the public good was multiplied, specifically, 1.2, 2, or 3.2) was small. Such variation is likely to have influenced individuals with different levels of prosociality in a different manner. Whereas a highly prosocial participant might have been influenced only a little by the context and therefore also cooperated in an “expensive” context such as Situation 2, a participant with a lower inclination towards prosociality might have been strongly influenced by the context and did not contribute when the multiplier was small, as in Situation 2. This notion was documented for instance by Isaac and Walker (1988), demonstrating that a decrease in the multiplier can lead to a significant increase in free-riding behavior. In a similar fashion to Situations 1 and 2, in Situation 3 we asked participants immediately after making their contribution decisions how many MUs they believed others had contributed on average. Situations 2 and 3 were presented in a counterbalanced order.

### Situation 4 (DG)

The participants played the role of a ‘dictator’. They were endowed with 10 MUs (5 CHF) and were asked to decide how many MUs to keep and how many to give to an anonymous recipient (one-shot). The participants were informed in advance that at the end of the study, their allocation was randomly assigned to another participant and implemented accordingly.

### Statistical Analyses

To capture the domain-general prosociality, we performed a factor analysis on participants’ prosocial behavior in the different situations. The contributions in the social dilemma situations were z-transformed and subjected to a principal axis factoring procedure. Factors based only on the common variance of the four situations were extracted from the original correlation matrix. The number of factors to be extracted was determined by the criterion wherein the eigenvalues were ≥ 1. The resulting factor scores were obtained using regressions. Since only one factor was extracted, we refer to this factor score as the ‘Domain-general Prosociality Index.’

The primary aim of this study was to examine whether domain-general prosociality can be explained by a task-independent neural trait measure. Thus, we conducted whole-brain voxel-wise Spearman’s rank correlations (separately for each frequency band) using the *Domain-general Prosociality Index* as the dependent variable. Correction for multiple testing was implemented by means of a nonparametric randomization approach (Nichols and Holmes 2002). The nonparametric randomization approach was used to estimate empirical probability distributions (number of randomizations used: 5000) and the corresponding corrected (for multiple comparisons) critical probability thresholds. For regions that displayed significant, whole-brain corrected correlations, the voxels with the strongest correlations (peak voxels) were then used for the construction of spherical regions of interest (ROIs; radius: 10 mm around the peak voxel). Mean current density within the ROIs was calculated and used for visualization and for additional regression analyses.

A conjunction analysis was carried out as a second approach to examine the neural traits underlying domain-general prosociality. Four whole-brain corrected Spearman’s rank correlation images between resting EEG and contribution decisions in the social dilemma situations were computed separately. A conjunction was then performed to quantify the overlap between the four correlations images.

## Results

### Behavioral Results

**Fig. 1:**
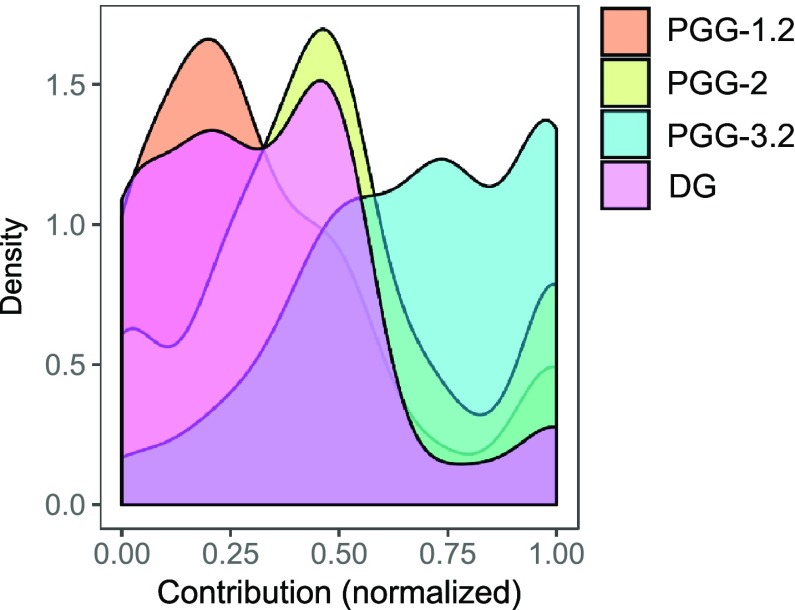
Kernel density distribution plots of the participants’ contribution in the four social dilemma situations (PGG-1.2, PGG-2, PGG-3.2 and DG)

As illustrated in Fig. 1, the four distributions of the participants’ contributions differed significantly between the different social dilemma situations (Wilcoxon signed rank tests: all six comparisons *p* < 0.001).

**Fig. 2:**
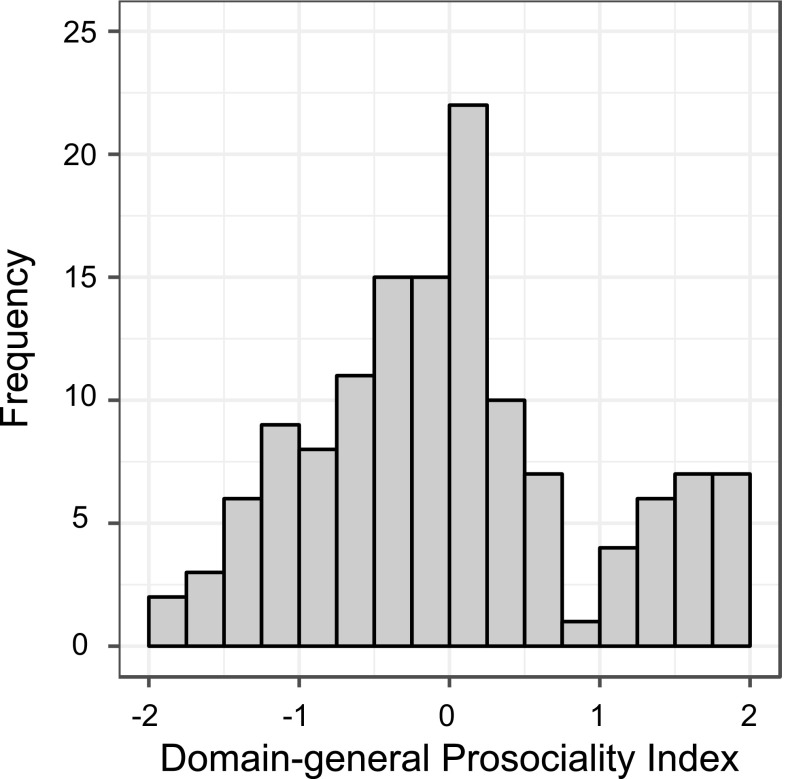
Histogram depicting the distribution of the *Domain-general Prosociality Index* among all participants

Factor analysis revealed an underlying structure composed of one single factor. The factor loadings were 0.92, 0.49, 0.60, and 0.31 for Situations 1–4 (that is, PGG-2, PGG-1.2, PGG-3.2 and DG), respectively. No other factor had an eigenvalue that exceeded 1 (eigenvalue of the next factor: 0.87). This factor, hereby referred to as ‘Domain-general Prosociality Index’, accounted for 49.9% of the variance in the four decisions. We observed large inter-individual differences in domain-general prosociality (Fig. 2). The *Domain-general Prosociality Index* varied from − 1.78 to 1.88 (*M* = 0, *SD* = 0.93). There was no correlation between the *Domain-general Prosociality Index* and the honesty-humility scale [*r*(131) = − 0.079, *p* = 0.37], nor with positive affect [*r*(131) = − 0.141, *p* = 0.11] or negative affect [*r*(131) = − 0.060, *p* = 0.50].

### Brain Results

**Fig. 3:**
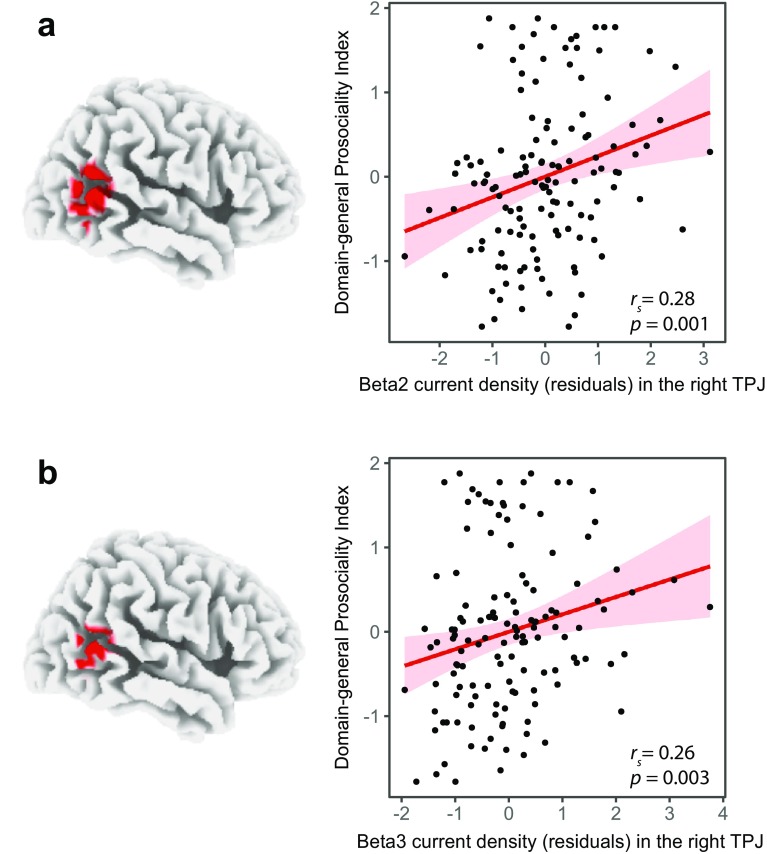
Relationship between resting beta current density and the *Domain-general Prosociality Index* in the right temporo-parietal junction. On the left, locations of the voxels that exhibited significant correlations (whole-brain corrected) in the beta 2 (**a**) and beta3 (**b**) frequency bands are indicated in red (*p* < 0.05). On the right, the scatter plots, based on a 10 mm spherical ROI around the corresponding peak voxels, demonstrate the positive association between the resting beta current density (residuals) and the *Domain-general Prosociality Index*, including regression lines (in red), and confidence intervals (95%)

To study the putative neural traits underlying individual differences in domain-general prosociality, we conducted whole-brain Spearman’s rank correlations with the *Domain-general Prosociality Index* as the dependent variable. Using sLORETA as a source localization technique to estimate the intracerebral sources underlying scalp-recorded resting EEG, we found that the *Domain-general Prosociality Index* was positively correlated with task-independent resting EEG in the beta2 and beta3 frequency bands. The results in the two beta bands were largely overlapping and comprised a cluster in the right temporo-parietal junction (TPJ, BAs 21, 22, 37, 39, and 40). Specifically, in the beta2 band, all 30 significant voxels fell into one cluster in the right TPJ (BAs 21, 22, 37, 39, and 40; MNI coordinates peak voxel: x = 60, y = − 60, z = 15, Fig. 3a). In the beta3 band, all 19 significant voxels fell into one cluster that also peaked in the right TPJ (BAs 21, 22, 37, 39, and 40; MNI coordinates peak voxel: x = 65, y = − 55, z = 5, Fig. 3b). Spearman’s rank correlations conducted with the two ROIs (spheres of 10 mm radius around the peak voxels) revealed positive correlation coefficients of *r*_*s*_(131) = 0.28, *p* = 0.001 in beta2 and *r*_*s*_(131) = 0.26, *p* = 0.003 in beta3.

Controlling for the belief regarding others’ contributions demonstrated that the correlation between resting beta current density in the TPJ and the *Domain-general Prosociality Index* remained significant: *r*_*s*_(130) = 0.21, *p* = 0.02 in beta2; *r*_*s*_(130) = 0.19, *p* = 0.03 in beta3 in the right TPJ.

Our findings were specific to the beta2 and beta3 frequency bands and the right TPJ, as no significant correlations were found in any other EEG frequency band, and in no other brain region was the resting beta2 or beta3 current density correlated with the *Domain-general Prosociality Index*.

**Fig. 4:**
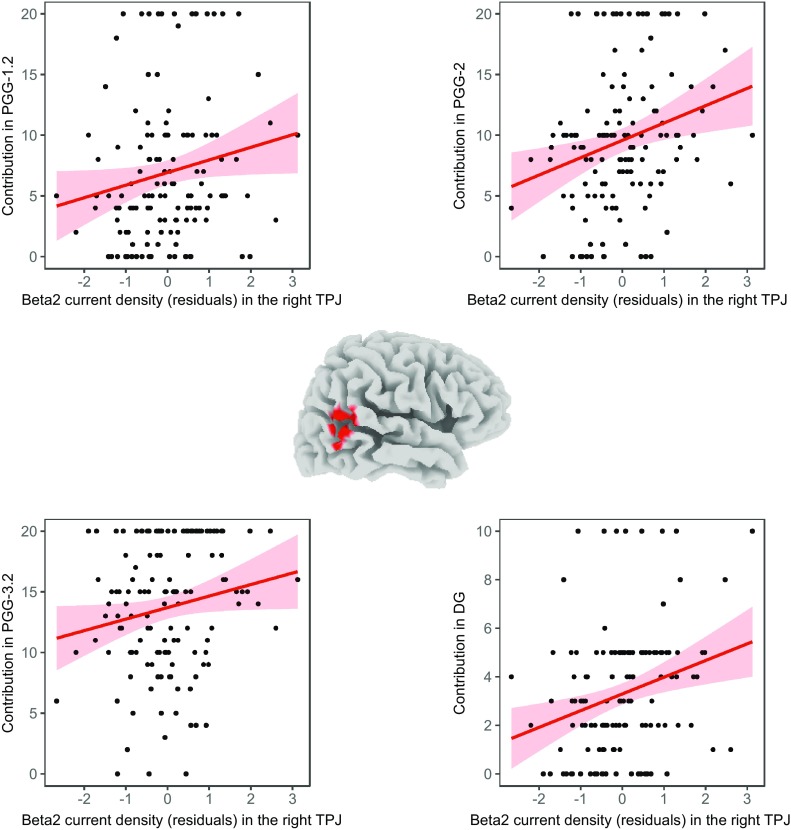
Conjunction analysis. In the center, the locations of all voxels that proved to be significant in the conjunction analyses for the four brain correlation images (whole-brain corrected) in the beta2 band is depicted. Red indicates *p* < 0.05. The scatter plots, based on a 10 mm spherical ROI around the corresponding peak voxel demonstrate the positive association between the resting beta2 current density (residuals) and the contributions in the different social dilemma situations. Regression lines and confidence intervals (95%) are indicated in red

Conjunction analysis confirmed the association between the resting beta current density in the right TPJ and the contributions in each of the four social dilemma situations. Specifically, in the beta2 band (Fig. 4), 23 significant voxels fell into one cluster in the right TPJ (BAs 21, 22, 39, and 40; MNI coordinates peak voxel: x = 60, y = − 55, z = 20). The Spearman’s rank correlations between resting beta2 current density and the contributions in the four social dilemma situations revealed positive significant correlation coefficients of *r*_*s*_(131) = 0.22, *p* = 0.006 in the PGG-1.2 (MNI coordinates of peak voxel: x = 65, y = − 45, z = 25), *r*_*s*_(131) = 0.27, *p* < 0.001 in the PGG-2 (MNI coordinates of peak voxel: x = 60, y = − 60, z = 15), *r*_*s*_(131) = 0.21, *p* = 0.008 in the PGG-3.2 (MNI coordinates of peak voxel: x = 65, y = − 50, z = 20), and *r*_*s*_(131) = 0.30, *p* < 0.001 in the DG (MNI coordinates of peak voxel: x = 60, y = − 55, z = 20). The four correlation coefficients (between resting beta2 current density in TPJ and PGG-1.2, PGG-2, PGG-3.2, DG) did not differ significantly, as demonstrated by the Meng’s test for comparing correlated non-parametric correlation coefficients (all *p* > 0.4).

In the beta3 band, eight significant voxels fell into one cluster in the right TPJ (BAs 21 and 22; MNI coordinates peak voxel: x = 65, y = − 55, z = 5).

## Discussion

In this study, we identified a specific neural trait marker—resting fast-wave oscillations originating from the temporo-parietal junction (TPJ)—for domain-general prosociality. More specifically, higher levels of beta2 and beta3 current density in the right TPJ were associated with higher prosocial contributions across different social dilemma situations.

Previous neural trait studies have revealed sources of individual differences for several domains of prosociality in different brain regions, for instance in the right TPJ (e.g., Morishima et al. 2012), dorsolateral prefrontal cortex (Knoch et al. 2010; Fermin et al. 2016), or anterior insula (Baumgartner et al. 2013; Watanabe et al. 2014). However, each of these studies has only focused on one single domain. In contrast to previous studies, we measured domain-general prosociality and investigated the neural signatures underlying an individual’s propensity to behave prosocially across different situations. We propose that resting EEG current density in the TPJ reflects an individual’s propensity to engage in prosocial behavior across situations. As confirmed by the conjunction analysis, prosocial decisions in all four social dilemma situations were indeed associated with higher resting beta current density in the TPJ.

To ensure that we obtained a pure measure of prosociality and to control for possible strategies, we asked our participants after their contribution decisions what they believed others contributed on average. Since the participants’ final pay-off also depended on the other participants’ contribution to the public good, the participants’ contribution decision could have been influenced by their belief of the other participants’ behavior. For example, a participant could strategically decide to contribute half of their endowment because they assumed that the other three players would contribute a similar amount. Another participant could contribute half of their endowment, according to their prosocial inclination, without strategically considering others’ decisions. Clearly, the same contribution of these two individuals does not indicate the same level of domain-general prosociality. To control for this possible confound, we ran additional analyses that accounted for the participants’ estimation of the others’ average contributions. The results of these additional analyses corroborated the finding that resting EEG current density in the right TPJ was significantly associated with pure domain-general prosociality.

One popular account of TPJ function posits that it allows attention to be shifted away from the self to focus on others’ perspectives (e.g., Lamm et al. 2016; for a recent review see; Steinbeis 2016) and to overcome one’s self-centered perspective (Hare et al. 2010; Strombach et al. 2015; Soutschek et al. 2016). As prosociality involves sacrificing resources for the benefit of others, prosocial decisions are likely to require a shift in attention away from the subject’s own state to focus on the needs of others. Interestingly, a lack in the capacity of distinction between the self and the others can indeed have deleterious effects on prosocial behavior (e.g., Decety and Lamm 2009). Furthermore, evidence from a modulation study suggests that the TPJ is causally involved in overcoming self-centeredness, rendering behavior prosocial (Soutschek et al. 2016). This leads to the speculation that people with higher resting beta current density in the TPJ, that is, people with higher abilities in self-other distinction and in overcoming self-centeredness, are more likely to exhibit higher levels of domain-general prosociality than their counterparts with lower abilities. Support for this speculation may also be found in online EEG studies that showed that beta oscillations originating from the TPJ play an important role when attempting to estimate other´s preferences (Park et al. 2018) or when differentiating between an ingroup or outgroup member (Riecansky et al. 2014).

Finally, we found that domain-general prosociality was not related to the honesty-humility scale of the HEXACO questionnaire. This result contrasts with previous studies that demonstrated a positive association between honesty-humility and different forms of prosociality (e.g., Hilbig et al. 2012; Aghababaei et al. 2014; Thielmann and Hilbig 2014). However, in these studies, prosociality was measured either with self-reports or with economic games that were entirely hypothetical and without financial consequences (however, see Zhao et al. 2016).

In sum, we believe that this is the first study to demonstrate that a specific neural trait marker—task-independent resting beta current density in the TPJ—explains individuals’ variation in domain-general prosociality. These results provide neural evidence as to why some people consistently behave more prosocially than others in different situations. The capacity to differentiate between the self and the others and to overcome self-centeredness might be a fundamental prerequisite for domain-general prosociality. A deeper understanding of the neural underpinnings of domain-general prosociality will help improving prosocial behavior, for instance in patients with autism spectrum disorder (a disorder characterized by a hypoactivation in the TPJ; e.g., Lombardo et al. 2011; Pantelis et al. 2015). However, with our results we cannot completely rule out the possibility that resting beta current density in the TPJ is not associated with behaviors that would still fall under the domain of prosociality, like for example, providing instrumental help or comforting.

Although neural traits are highly stable across time, they are not immutable. It has been well documented that repeated practice of skills as well as techniques such as neurofeedback or meditation have the capacity to induce permanent changes in neural structure or function (e.g., Lazar et al. 2005; Takeuchi et al. 2010; Ghaziri et al. 2013). The results of our study could contribute to the improvement of treatment precision via tomographic neurofeedback (e.g., Congedo et al. 2004; Liechti et al. 2012) in order to specifically target certain EEG oscillations originating from the TPJ.
